# A controlled, parallel, cluster-randomized trial of community-wide screening and treatment of asymptomatic carriers of *Plasmodium falciparum* in Burkina Faso

**DOI:** 10.1186/1475-2875-12-79

**Published:** 2013-02-27

**Authors:** Alfred B Tiono, Alphonse Ouédraogo, Bernhards Ogutu, Amidou Diarra, Sam Coulibaly, Adama Gansané, Sodiomon B Sirima, Gregory O’Neil, Amitava Mukhopadhyay, Kamal Hamed

**Affiliations:** 1 Centre National de Recherche et de Formation sur le Paludisme, Ouagadougou, Burkina Faso; 2 Centre for Clinical Research, Kenya Medical Research Institute, Nairobi, Kenya; 3 Novartis Pharma AG, Basel, Switzerland; 4 Novartis Healthcare Private Limited, Hyderabad, India; 5 Novartis Pharmaceuticals Corporation, East Hanover, NJ, USA

**Keywords:** Malaria, *Plasmodium falciparum*, Asymptomatic carriers, Mass screening, Transmission, Artemether-lumefantrine

## Abstract

**Background:**

In malaria-endemic countries, large proportions of infected individuals are asymptomatic, constituting a reservoir of parasites for infection of newly hatched mosquitoes. This study evaluated the impact of screening and treatment of asymptomatic carriers of *Plasmodium falciparum*.

**Methods:**

Eighteen villages were randomized (1:1) to study arms and inhabitants participated in four community screening campaigns: three before the rainy season ~1 month apart, and the fourth after the rains at ~12 months. On day 1 of campaigns 1–3, asymptomatic carriers in the intervention arm were identified by rapid diagnostic test and treated with artemether-lumefantrine. Outcomes were symptomatic malaria with parasite density >5,000/μL per person-year in children < 5 years and change in haemoglobin between days 1 and 28 of campaign 1.

**Results:**

At 12 months, the number of symptomatic malaria episodes with a parasite density >5,000/μL per person-year in children < 5 years was not significantly different between arms (1.69 *vs* 1.60, p = 0.3482). Mean haemoglobin change in asymptomatic carriers during campaign 1 was greater in the intervention *vs* control arm (+0.53 g/dL *vs* -0.21 g/dL, p < 0.0001). ANCOVA demonstrated that mean asymptomatic carriage at the cluster level was lower in the intervention *vs* control arm at day 1 of campaigns 2 (5.0% *vs* 34.9%, p < 0.0001) and 3 (3.5% *vs* 31.5%, p < 0.0001), but showed only a small difference at day 1 of campaign 4 (34.6% *vs* 37.6%, p = 0.2982). Mean gametocyte carriage was lower in the intervention *vs* control arm at day 1 of campaigns 2 and 3 (0.7% *vs* 5.4%, p < 0.0001; 0.5% *vs* 5.8%, p < 0.0001), but was similar at day 1 of campaign 4 (4.9% *vs* 5.1%, p = 0.7208).

**Conclusions:**

Systematic screening and treatment of asymptomatic carriers at the community level did not reduce clinical malaria incidence in the subsequent transmission season, indicating greater levels of parasite clearance are required to achieve a sustained impact in this setting.

## Background

In malaria-endemic countries, a large proportion of *Plasmodium falciparum* infections are asymptomatic. Microscopy-detected levels of asymptomatic carriage as high as 39% have been reported [1-3] but the exact role of asymptomatic carriers in the dynamics and transmission of malaria is currently unclear [4]. However, as they do not seek treatment for their infection, asymptomatic carriers can have high levels of gametocytes [1] and constitute an important parasite reservoir available for infection of newly hatched mosquitoes [4,5]. Even in areas of highly seasonal malaria transmission, studies have shown that a considerable proportion of the population remain positive for parasitaemia throughout the year [1,3]. In addition to constituting a parasite reservoir responsible for malaria transmission, individuals with asymptomatic parasitaemia are at risk of developing anaemia and may progress to symptomatic malaria.

The systematic identification and treatment of asymptomatic carriers could potentially reduce disease transmission by reducing the pool of parasites carried by these individuals. This hypothesis is supported by a modelling and simulation analysis that evaluated the impact of community screening campaigns followed by treatment of asymptomatic carriers with artemether-lumefantrine (AL) (Coartem®, Novartis Pharma AG, Basel, Switzerland) [6]. This simulation suggested campaign visits placed in close succession in the period prior to the malaria transmission season should effectively clear asymptomatic infection from communities and significantly reduce the subsequent incidence of disease in those communities [6]. Similar predictions were obtained by Okell *et al*. in their modelling of the impact of two mass treatment strategies [7].

AL has demonstrated consistently high efficacy and safety for over a decade and is included on the WHO Model List of Essential Medicines [8,9]. For the intervention to be effective, a large proportion of asymptomatic carriers in a defined district need to receive treatment to significantly reduce the reservoir of parasites in that community. For this reason the unit of randomization and analysis used in this study was the village. This study, therefore, evaluated the impact on malaria incidence of community-level screening (using RDT) and treatment of asymptomatic carriers of *P*. *falciparum* on the number of microscopy-confirmed cases of symptomatic malaria with a parasite density >5,000/μL seen over a 12-month period, compared with no screening with RDT nor treatment of asymptomatic carriers, as well as the direct benefit from treatment on haemoglobin (Hb) levels in asymptomatic carriers who received AL. Subjects in both the intervention and control arms had blood smears taken for delayed microscopy reading. The parasite density requirement was used to avoid a potential anti-conservative bias. If febrile patients with lower levels of parasitaemia were included in the analysis, then participants in the control arm with non-malarial fever might incorrectly be counted as symptomatic malaria cases, making the intervention appear more effective. Data showed systematic screening and treatment of asymptomatic carriers in this setting did not reduce clinical malaria incidence in the subsequent transmission season, possibly indicating greater levels of parasite clearance are required to achieve a sustained impact. Hb levels were, however, improved with the intervention.

## Methods

### Study population

The study took place in the health district of Saponé, about 50 km south of Ouagadougou in Burkina Faso, which is an area with marked seasonal malaria transmission of *P*. *falciparum* from June to November [10]. All clusters considered for selection were part of a demographic surveillance system that routinely monitors the vital events occurring within these villages (e.g., births, deaths, in/out migration). All inhabitants of each cluster were invited to participate in the trial. Written informed consent was obtained from all study participants or their legal guardians.

### Study design

**Figure 1:**
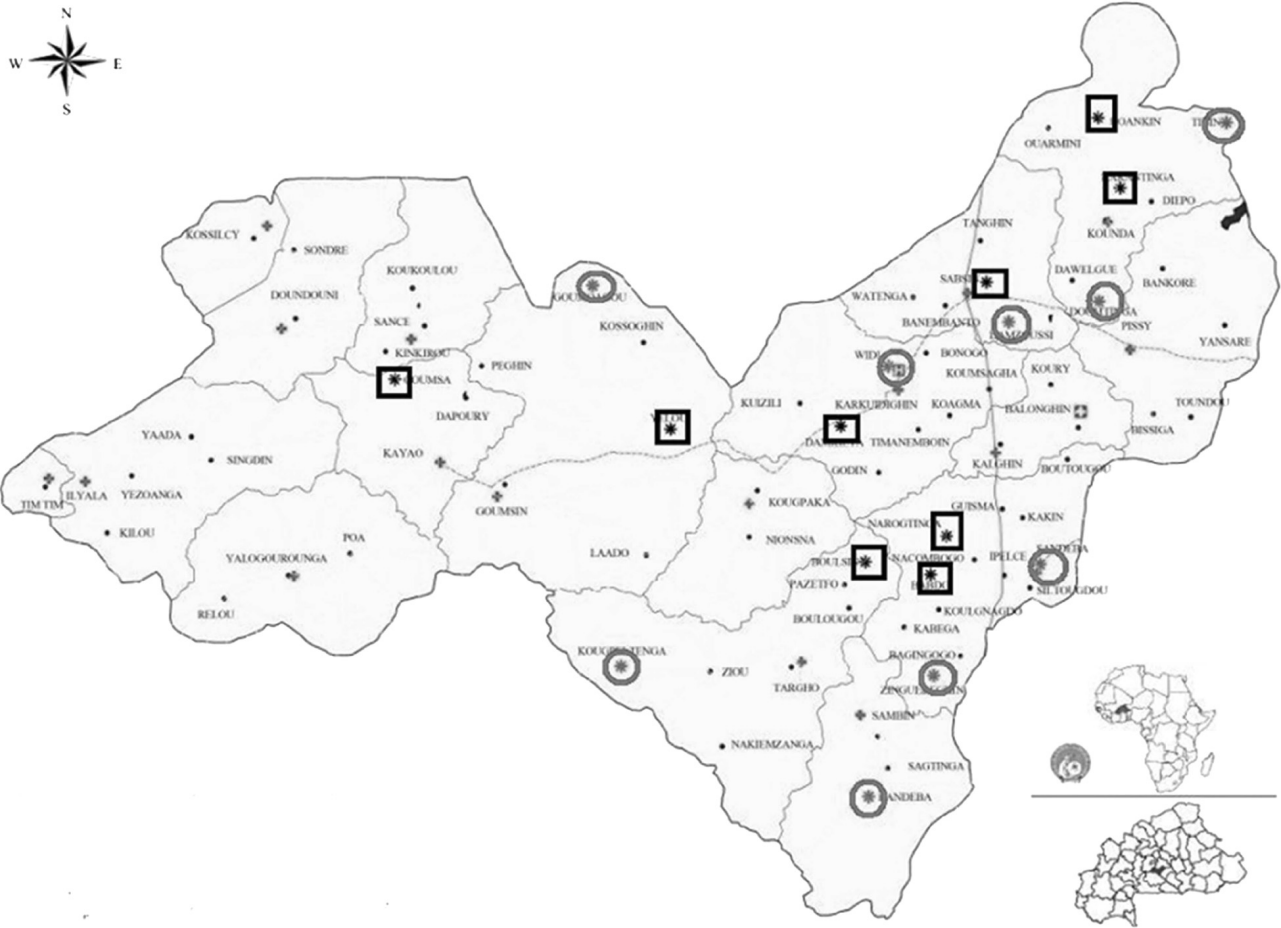
**Map of the Saponé demographic surveillance system area.** The control clusters are represented by circles whereas intervention clusters are represented by squares.

**Figure 2:**
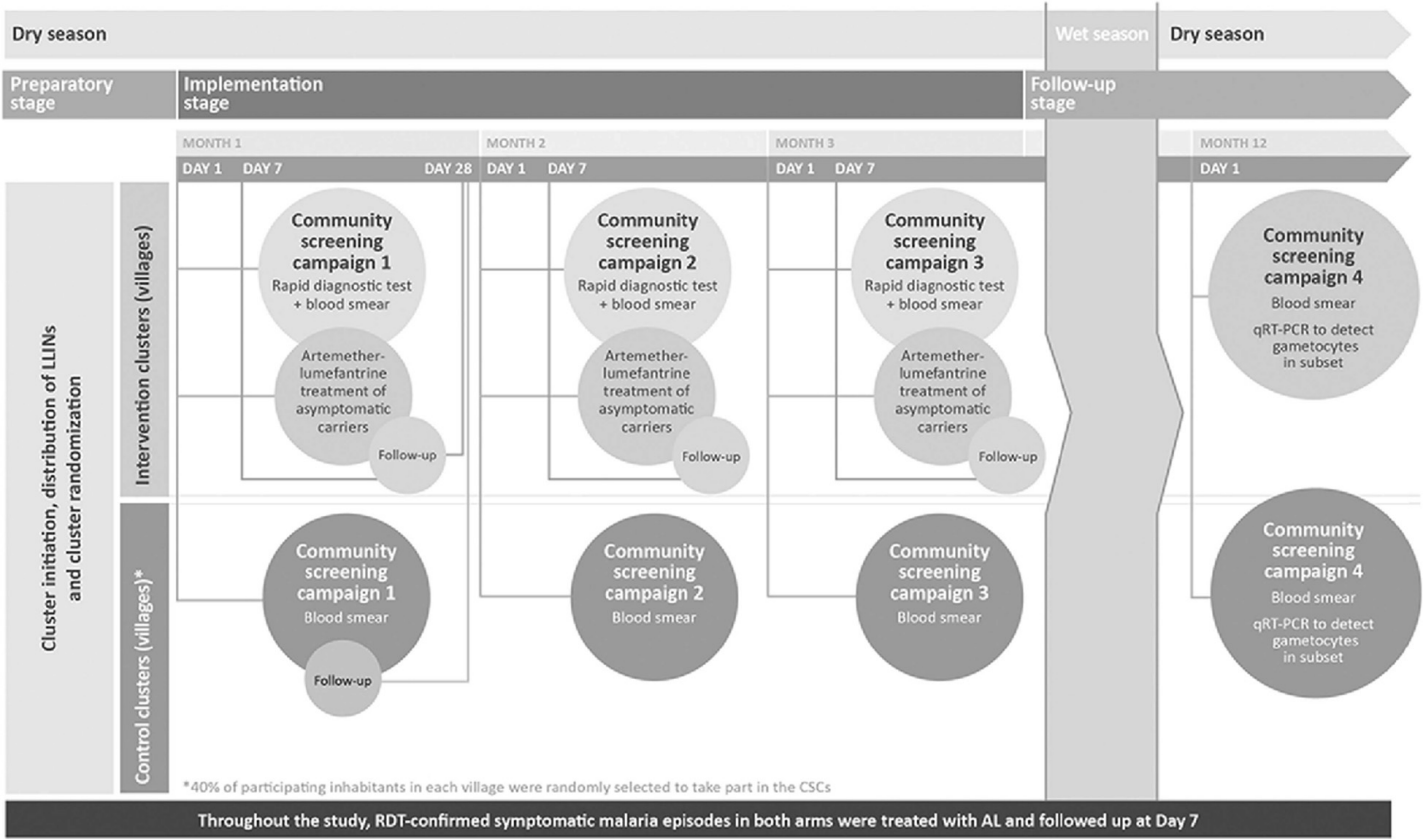
Study design.

This study was a single-centre, controlled, parallel, cluster-randomized study to evaluate the effect of systematic treatment of *P*. *falciparum* asymptomatic carriage at a community level on the number of symptomatic malaria episodes, with a parasite density >5,000/μL per person-year in children < 5 years of age over a 12-month period compared with no treatment of asymptomatic carriers. Eighteen clusters, each comprised of one village, reporting to eight local health facilities were selected for inclusion in the trial as shown in Figure 1. The study consisted of three phases (preparatory, implementation, and follow-up) as shown in Figure 2.

### Cluster randomization procedure

Clusters were randomized into either the intervention or control arm following a 1:1 ratio at a public draw involving the investigator, representatives from the sponsor, and the leaders from every cluster. Since several clusters reported to the same local health facilities, the local health facility was used as a stratification factor to ensure a balanced distribution of clusters between study arms. Thus, seven envelopes (numbered 1 to 7) were prepared by the trial statistician. Each contained the name of a single local health facility and the relevant clusters, as well as capsules each containing the name of a single cluster. The envelopes were placed on a table and sorted by number (number 1 on top down to number 7). Envelopes were opened sequentially from the lowest to the highest number. When each envelope was opened, the name of the local health facility and its clusters were read to the audience, and all capsules from within the envelope were placed in a transparent urn. A representative from one of the clusters listed in the envelope was selected for the drawing of the capsules. When a capsule was drawn, it was handed over to the conductor of the session who opened it and presented the name of the cluster it contained to the audience. The cluster was assigned to the control or intervention arm in an alternating fashion, i.e. the first cluster drawn from the first envelope was assigned to the control arm, the cluster drawn second to the intervention arm, until the 18^th^ cluster was drawn from the seventh envelope and assigned to the intervention arm.

### Study procedures

During the implementation phase of the study, intervention and control village inhabitants participated in three community screening campaigns that included home visits by mobile teams of health care professionals. A fourth campaign was conducted after the rainy season had ended. This campaign marked the end of the study at ~12 months. At these visits, finger-prick blood samples were taken from the entire study population in the intervention arm and a randomly selected 40% subset of the control arm. In the intervention arm, the population was screened on day 1 of campaigns 1–3 using RDT (First Response® Malaria Ag, Premier Medical Corp Ltd., Nani-Daman, India). Those individuals with a positive test result received treatment with AL/AL Dispersible. Subjects with positive RDT in the intervention arm also had microscopy performed on day 7 of campaigns 1–3 and day 28 of campaign 1. To assess parasitological clearance, microscopy with delayed reading, to ensure that study personnel and screened control subjects remained unaware of their status, was performed on day 1 of each campaign in both arms. Blood was also taken for Hb assessment on day 1 and day 28 of campaign 1 and day 1 of campaign 4. Subjects with symptoms indicative of malaria were requested to attend their local health facility for confirmatory testing.

Following campaign 3, study participants in both arms were followed up for passive detection of symptomatic malaria throughout the wet season when malaria transmission is high. Participants were encouraged to report to their local health care facility or clinic as soon as they felt unwell. An RDT was performed for all participants attending the local health facility with confirmed fever (axillary temperature ≥37.5°C) or history of fever within the last 24 hours. A blood smear was also taken. Each episode of symptomatic malaria was treated and the patient followed up at day 7. Parasitological cure was assessed by microscopy for each symptomatic malaria episode on day 7.

On day 1 of campaign 4, a blood sample was also collected for gametocyte assessment by quantitative reverse transcription-polymerase chain reaction (qRT-PCR). qRT-PCR was conducted in 1,999 randomly selected subjects drawn from the entire intervention group and a 40% subgroup of the control population.

Adverse events (AEs) were collected during a period of 7 days from treatment administration, and serious adverse events (SAEs) were recorded from the time of consent until 30 days after the subject had stopped study participation (defined as time of last dose of investigational/alternative treatment taken or last visit, whichever was later).

### Study treatments

During the study, all individuals with a positive RDT test in any arm, either with malaria symptoms or not, received AL/AL dispersible (20 mg artemether and 120 mg lumefantrine) twice a day for three consecutive days. The first dose was supervised and treatment was adjusted according to body weight: as follows: 5 to <15 kg: 1 × AL dispersible tablet b.i.d.; 15 to <25 kg: 2 × AL dispersible tablets b.i.d.; 25 to <35 kg: 3 × AL tablets b.i.d.; ≥35 kg: 4 × AL tablets b.i.d.

Any female > 8 years of age with a positive RDT for symptomatic malaria who did not take the urine pregnancy test or who was in the first trimester of pregnancy received alternative treatment to AL, according to national guidelines, as did individuals with contra indications to AL and AL dispersible. When alternative treatment was administered to a subject this subject had to follow the same assessments as a subject who received AL.

Neither RDT nor treatment of asymptomatic carriers was performed on day 1 of campaign 4, but subjects who presented with symptomatic malaria were treated and followed up. In cases of deterioration or severe malaria, the patient was referred to the district hospital. Use of prescription malaria treatments other than AL was tabulated but no record was taken of any over-the-counter purchases of anti-malarials.

Throughout the study, community health care workers visited households to check and document treatment adherence of asymptomatic carriers and those with symptomatic malaria through the use of a drug accountability log and tablet counts.

The use of Olyset® (Sumitomo Chemical Co, Ltd, Tokyo, Japan) long-lasting, insecticide-impregnated bed nets (LLINs), which were provided to all participants who gave informed consent before the implementation phase, was checked at the home visits conducted at least every two months.

### Laboratory methods

The blood films obtained during visits and for symptomatic malaria assessment were air-dried and Giemsa-stained for examination. Examination was done using a light microscope fitted with a 100 X oil immersion lens at a single laboratory for all clusters. At least 200 thick film fields were examined before a slide was declared negative. If asexual forms of *Plasmodia* were found, a total of 200 thick film fields were screened for *Plasmodium* species other than *P*. *falciparum*. When *P*. *falciparum* was present, a count of the asexual forms against leukocytes was made using a tally counter. Counting was done based on at least 200 leukocytes according to the WHO standards. If less than 10 parasites were identified from the 200 leukocyte screen, counting was extended to 1,000 leukocytes. If *P*. *falciparum* gametocytes were seen, a gametocyte count was performed against 1,000 leukocytes. All slides were read by two independent microscopists. If the ratio of densities from the first two readings was >1.5 or < 0.67 (or if less than 30 parasites were counted with an absolute difference of more than 10 in the number of parasites), the slide was evaluated by a third microscopist. The definitive result was the mean of the parasite density of the two most concordant reading results. Microscopist competency was evaluated twice a year by proficiency testing, in which a set of 20 slides is provided to each microscopist for reading. Only those graded as ‘competent’, with a score of at least 80%, were involved in the reading of slides.

qRT-PCR was performed at the Swiss Tropical and Public Health Institute (Basel, Switzerland) using whole blood collected from the participant’s fingertip. After blood collection into EDTA coated microcontainer tubes, 50 μL of blood were transferred immediately into tubes preloaded with 250 μL RNAprotect solution (Qiagen, Valencia, CA, USA) and stored at 4°C until material was shipped to the central genotyping laboratory. One hundred and fifty μL of blood/RNAprotect solution were processed for quantification of gametocytes using the RNeasy Plus 96 kit (Qiagen) according to the manufacturer’s protocol with an additional on column step using the RNase-free DNase Set (Qiagen). RNA was eluted in 50 μL H_2_0 and 2 μL were used each for qRT-qPCR of *P*. *falciparum* A-type 18S RNA and *P*. *falciparum* pfs25 transcripts using the TaqMan RNA-to-Ct 1-Step Kit (Applied Biosystems, Carlsbad, CA, USA). A qPCR was performed to demonstrate absence of genomic DNA using the TaqMan gene expression Master Mix (Applied Biosystems). Ct-values were converted into pfs25 templates/μL using standard curves generated with cloned amplicons, and subsequently transcript copy numbers were converted into gametocytes/μL by diluting visually quantified gametocytes from *P*. *falciparum* strain 3D7.

Hb level was measured using the HemoCue® Hb 201+ rapid test (Ängelholm, Sweden) using blood collected by finger-prick on day 1 and day 28 of campaign 1 and on day 1 of campaign 4.

### Statistical methods

#### Data analysis

Analyses in this study were focused on cluster level data such that the cluster was considered as an experimental unit and observations were based on individual subjects in each cluster. The analysis was performed in two stages. In the first stage, a summary measure was obtained for each cluster and in the second stage a comparison was performed based on two sets of cluster-specific measurements. Cluster level data were summarized using summary statistics of cluster means for continuous variables and cluster percentages for categorical variables. Individual level data were also reported using summary statistics for continuous variables and number with percentage for categorical variables [11].

One-sided t-tests of equal means for study endpoints were conducted to a significance level of 0.05 based on cluster-level summary data. These independently assessed endpoints were the mean of cluster means of number of symptomatic malaria episodes at a parasite density of >5,000/μL per person-year in children < 5 years (at day 1 in campaign 1 and in the post-campaign follow-up period) and change in Hb level (g/dL) from day 1 to day 28 of campaign 1 in asymptomatic carriers > 6 months of age (at day 1 and day 28 of campaign 1).

Analyses of covariance (ANCOVA) were performed for prevalence of microscopy-confirmed gametocyte carriers and prevalence of microscopy-confirmed asymptomatic carriers of *P*. *falciparum* at day 1 of campaign 4. These were assessed during the post-campaign follow-up period (i.e. the day after day 7 of campaign 3 up to and including the day after the last study visit. Age was considered to be the age on day 1 of campaign 1. Cluster level prevalence of microscopy-confirmed asymptomatic carriage at campaigns 2, 3 and 4 was analysed by fitting an ANCOVA model with study arm as factor and prevalence of asymptomatic carriers at campaign 1 as a covariate. Prevalence of gametocyte carriage at campaigns 2, 3 and 4 was also analysed by fitting an ANCOVA model with study arm as factor and prevalence of gametocyte carriage at campaign 1 as covariate.

Hb levels at day 1 and day 28 of campaign 1 were compared and age was considered to be that at the time of the assessment. Mean Hb levels at campaign 4 in children aged > 6 months up to < 5 years were analysed by fitting an ANCOVA model with study arm as factor and the corresponding campaign 1 summary measure as a covariate.

Data were presented by treatment (AL/AL dispersible) and further broken down by campaign visit. Complete listings of medication used (AL/AL dispersible or alternative treatment) were generated.

All hypothesis testing was conducted one-sided at a 0.05 level of significance and all confidence intervals were reported based on two-sided 90% confidence intervals. No adjustments for multiplicity were performed.

#### Handling of missing values/censoring/discontinuations

The denominator used to calculate incidence of symptomatic malaria episodes at a parasite density >5,000/μL in the cluster population was corrected for cluster subjects who were not observed for the whole study duration (emigration, immigration, birth, death, etc.). If reporting failed at a large scale in any cluster, the cluster was to be excluded from the incidence of symptomatic malaria analysis for evaluable clusters.

Hb level was assessed in subjects with both day 1 and 28 measurements from campaign 1. In the intervention clusters, the initiation of treatment and the conduct of the day-28 assessment was based on a positive RDT result.

#### Ethics section

The protocol and the proposed informed consent form were reviewed and approved by the Centre National de Recherche et de Formation sur le Paludisme Institutional Review Board and by the National Ethical Committee for Health Research of Burkina Faso. Prior to study initiation, a community meeting was held in each of the selected clusters to discuss the study with the community. The freedom of each individual household and a household member to decide on participation was discussed to minimize the potential influence of key opinion leaders in each cluster. Individual informed consent was obtained from each participant during a visit to the household before any study procedure.

## Results

### Study demographics and baseline characteristics

**Figure 3:**
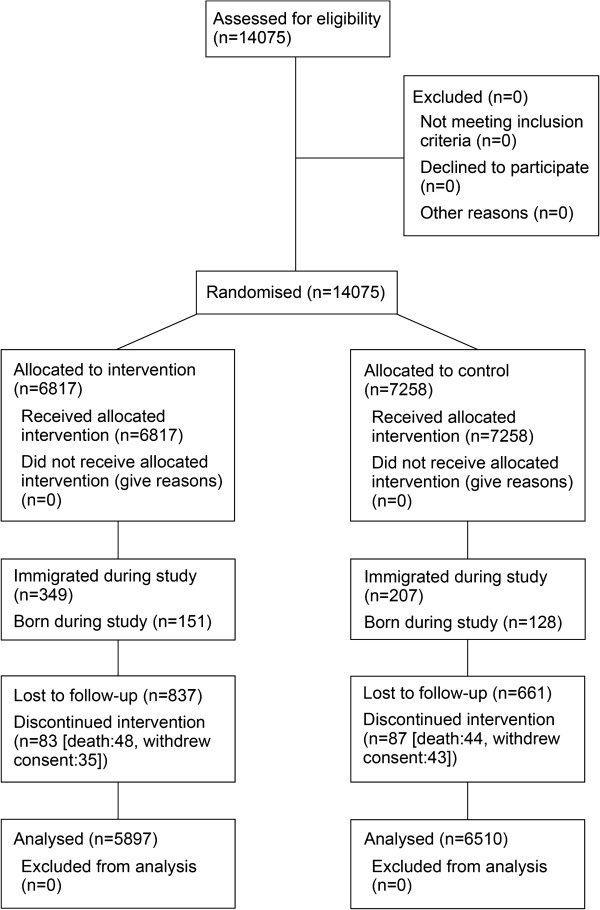
Flow chart showing numbers of participants throughout the study – trial profile.

A total of 6,817 persons in the intervention arm and 7,258 persons in the control arm were recruited and enrolled in this study, as shown in Table 1. The intervention and control arms were comparable in terms of demographic characteristics with the exception of ethnicity; the intervention arm had a higher proportion of Fulani. At the individual level, the intervention and control arms were comparable in terms of mean percentage of subjects completing the study, and were similar with respect to births after cluster initiation (Figure 3). Immigration was more common in the intervention arm. Discontinuation due to death or withdrawn consent occurred at similar rates, loss to follow up (the most common reason for discontinuation) was slightly more common in the intervention arm.

**Table 1:** Study population baseline demographics – individual-level data

**Characteristic**	**Intervention arm**	**Control arm**
**Number enrolled**	**6,817**	**7,258**
**Born after cluster initiation (%)**	**151 (2.2)**	**128 (1.8)**
**Immigrant during the study**	**349 (5.1)**	**207 (2.9)**
**Persons completed**	**5,897 (86.5)**	**6,510 (89.7)**
**Male sex (%)**	46.4	47.4
**Age at CSC1 day 1**		
**Mean (years)**	24.1	23.4
**Age group (%)**		
≤6 months	2.1	1.5
>6 months to <5 years	14.9	14.2
5 to 9 years	15.8	16.6
10 to 14 years	15.5	15.3
≥15 years	51.7	52.5
**Ethnicity (%)**		
Mossi	90.6	95.7
Fulani	8.7	3.8
Bissa	0.01	0.03
Gourounsi	0.03	0.2
Other	0.7	0.3

Compliance with LLIN was good at the household checks conducted at least twice monthly, as was AL adherence in those identified as asymptomatic carriers and in individuals with symptomatic malaria.

### Incidence of symptomatic malaria episodes

**Figure 4:**
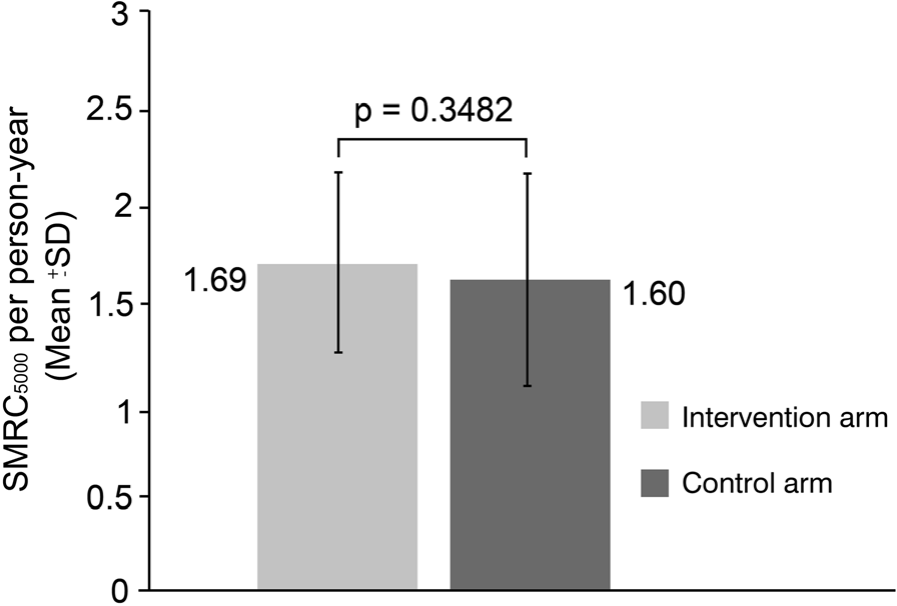
Symptomatic malaria episodes with a parasite density >5,000/μL (SMRC5000) per person-year in children aged < 5 years.

At 12 months, the number of RDT and microscopy-confirmed symptomatic malaria episodes with a parasite density >5,000/μL per person-year in infants and children aged < 5 years was not significantly different between the intervention and control arms (1.69 [SD 0.436] *vs* 1.60 [SD 0.526]; p = 0.3482; Figure 4). The number of symptomatic malaria episodes of any parasite density per person-year in infants and children aged < 5 years was also not significantly different between the two arms.

### Haemoglobin levels in asymptomatic carriers

Microscopy-confirmed asymptomatic carriers in intervention clusters showed mean increases in Hb level compared with the decreases seen in asymptomatic carriers in control clusters (Table 2). The between-arm difference was statistically significant at the cluster level although not sufficiently large to be clinically meaningful.

**Table 2:** Cluster-level data change in haemoglobin level (g/dL) in asymptomatic carriers >6 months of age from day 1 to day 28 of campaign 1 by study arm

	**Intervention arm**			**Control arm**			**p value**
	**Campaign 1**			**Campaign 1**			
	**Day 1**	**Day 28**	**Change**	**Day 1**	**Day 28**	**Change**	
	**(n = 2,387)**	**(n = 2,116)**		**(n = 1,136)**	**(n = 1,091)**		
Mean (SD)	11.81 (0.329)	12.33 (0.318)	0.53 (0.256)	12.06 (0.345)	11.86 (0.373)	-0.21 (0.266)	< 0.0001

n = Number of subjects from all clusters.

### Incidence of asymptomatic parasitaemia and gametocyte carriage

**Figure 5:**
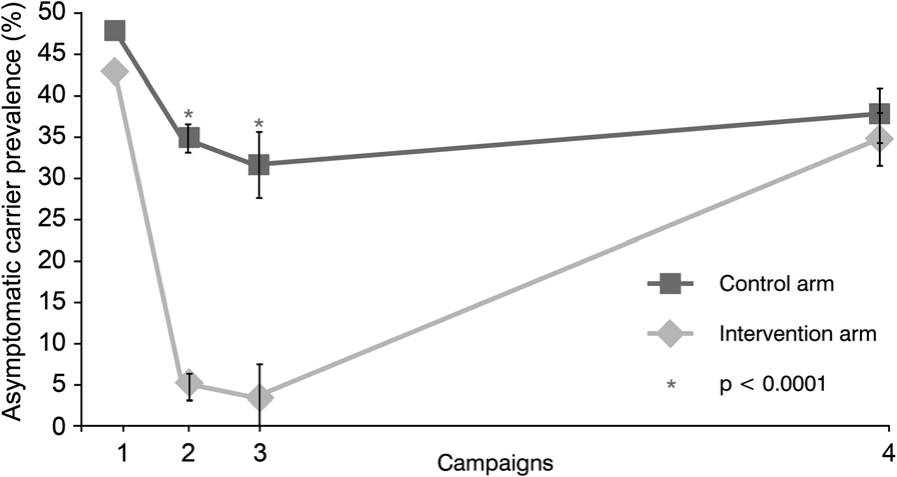
ANCOVA results for prevalence of microscopy-confirmed asymptomatic carriers.

ANCOVA results for the prevalence of asymptomatic parasitaemia at the cluster level revealed lower prevalence in the intervention arm than in the control arm at day 1 of campaign 2 (5.0% *vs* 34.9%; p < 0.0001) and day 1 of campaign 3 (3.5% *vs* 31.5%; p < 0.0001), but showed only a small difference at day 1 of campaign 4 at the end of the study (34.6% *vs* 37.6%; p = 0.2982; Figure 5). Mean asexual parasite density was higher in the intervention arm than the control arm at day 1 of campaign 3 (5,347.4/μL *vs* 1,062.3/μL; p = 0.0204) but not significantly different at any other time point. Recorded mean asexual parasite densities at any campaign ranged from 1,074/μL to 5,347/μL in the intervention arm and 1,062/μL to 2,289/μL in the control arm.

**Figure 6:**
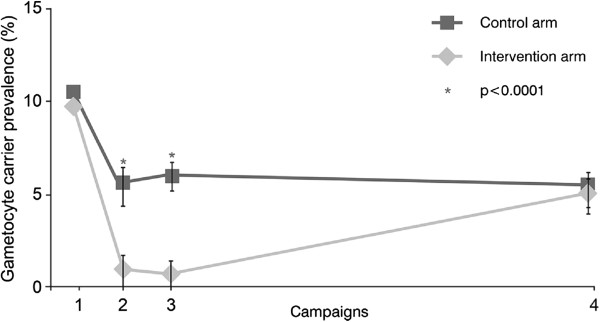
ANCOVA results for prevalence of microscopy-confirmed gametocyte carriers.

ANCOVA results for the prevalence of gametocyte carriage at the cluster level showed lower prevalence of gametocyte carriage in the intervention arm than the control arm at day 1 of campaigns 2 and 3 (0.7% *vs* 5.4%; p < 0.0001 and 0.5% *vs* 5.8%; p < 0.0001), but little difference at day 1 of campaign 4 at the end of the study (4.9% *vs* 5.1%; p = 0.7208; Figure 6). Prevalence of gametocytes at day 1 of campaign 4 as assessed by qRT-PCR was around 8-fold greater in both arms compared with microscopy (49.7% *vs* 6.0% intervention; 47.3% *vs* 5.4% control). Mean gametocyte density did not significantly differ between the intervention and control arms at any time point. Recorded mean gametocyte densities at any campaign ranged from 39.2/μL to 48.6/μL in the intervention arm and 21.6/μL to 49.7/μL in the control arm.

### Safety evaluation

A summary of the incidence of AEs and SAEs is presented in Table 3. Overall, there were no notable differences in AEs or SAEs between the intervention arm and the control arm at either the cluster or individual level and no new or unexpected safety findings were recorded. In total, 0.3% of treated asymptomatic carriers reported at least one AE within 7 days of starting treatment. The majority of deaths in the study occurred in subjects who had received no study medication. Analysis of death rates in the intervention and control arms revealed no statistically significant differences for total deaths or deaths due to malaria.

**Table 3:** Number (%) of adverse events and serious adverse in artemether-lumefantrine-treated subjects

	**Adverse events up to day 7**		**Serious adverse events up to day 30**	
	**AL-treated asymptomatic carriers**	**AL-treated with symptomatic malaria episode with >5,000/μL parasites**	**AL-treated asymptomatic carriers**	**AL-treated with symptomatic malaria episode with >5,000/μL parasites**
	**(n = 3,819)**	**(n = 2,554)**	**(n = 3,819)**	**(n = 2,554)**
Abdominal pain	1 (0.03%)	-	1 (0.03%)	-
Nausea	1 (0.03%)	-	1 (0.03%)	-
Vomiting	1 (0.03%)	14 (0.5%)	-	21 (0.8%)
Death	1 (0.03%)	-	1 (0.03%)	-
Pyrexia	1 (0.03%)	-	1 (0.03%)	-
Malaria	5 (0.1%)	2 (0.08%)	4 (0.1%)	5 (0.2%)
Bronchitis	2 (0.05%)	-	2 (0.05%)	-
Pneumonia	2 (0.05%)	1 (0.04%)	2 (0.05%)	2 (0.08%)
Respiratory tract infection	1 (0.03%)	-	1 (0.03%)	-
Dizziness	1 (0.03%)	-	1 (0.03%)	-
Asthmatic crisis	1 (0.03%)	-	-	-
Somnolence	-	-	-	1 (0.04%)

## Discussion

In this study, community screening and targeted treatment of asymptomatic carriers of *P*. *falciparum* did not have a significant impact on the number of episodes of symptomatic malaria with a parasite density >5,000/μL per person-year in infants and children aged < 5 years. Although the primary end point relating to disease burden was not met, the declines in asymptomatic parasitaemia and gametocyte carriage in the intervention arm at day 1 of campaigns 2 and 3 show that treatment of asymptomatic carriers with AL did reduce the parasite reservoir. This effect, however, was not sustained through the subsequent wet season to day 1 of campaign 4 and did not translate into a reduction in symptomatic malaria episodes in children. The observed effect therefore was not high enough to interrupt malaria transmission. These results do not reflect those indicated by the earlier simulation analysis [6]. There are likely a variety of reasons for this difference, including the dilution effect via infected vectors from surrounding villages not involved in the study and the high transmission intensity.

The observed levels of gametocyte carriage by microscopy in the intervention arm were reduced to 0.4% at day 1 of campaign 3. However, at day 1 of campaign 4 the qRT-PCR results for gametocyte carriage revealed levels of carriage 8-fold greater than indicated by microscopy. These findings suggest that by using RDT and subsequent microscopic confirmation a notable proportion of gametocyte carriers were undetected. One recent study has suggested that significant proportions (up to 50% in low-endemic settings) of all human-to-mosquito transmissions are caused by submicroscopic parasite carriage [12]. If a proportion of carriers were indeed not treated, this may explain the absence of a sustained reduction in transmission seen in this study. A study of mass drug administration with sulphadoxine-pyrimethamine in The Gambia concluded that only a small number of *P*. *falciparum* carriers are required to start the next transmission season and the results of this study support this conclusion [13]. As discussed by Bousema and Drakely, the use of PCR to screen the intervention population would have led to much higher levels of asymptomatic carrier detection and subsequent clearance of gametocytes, perhaps to a level sufficient to begin to impact disease transmission [14]. However, such an intensive screening process could not be considered a practical public health control option with the currently available technology. RDTs likely still have a role to play in large-scale malaria interventions. For example, they have been used to detect local foci of asymptomatic infection in Zambia [15]. Administration of treatment to asymptomatic carriers surrounding index cases forms the basis of the ‘focal screening and treatment’ strategy for malaria control and elimination [16].

Overall, 96.1% of the population in the intervention arm who consented to participation were tested by RDT. It is unlikely that a higher level of coverage could be achieved during a routine public health campaign. Even if complete coverage of all inhabitants of a community could be achieved, it is impossible to prevent the introduction of disease by population migration and the highly mobile mosquito vector. Indeed, the close proximity of the intervention clusters to over 60 villages that were not included in the study make the backflow of infected vectors highly likely. This dilution of the intervention could have been minimized by extending the screening and treatment of asymptomatic carriers to villages surrounding the intervention clusters, but excluding these individuals from the evaluation of the trial end points.

The decrease from baseline in prevalence of asymptomatic parasitaemia and gametocyte carriage between day 1 of campaigns 1 and 4 in both the intervention and control arm suggests a possible study effect owing to availability of AL for all confirmed cases of malaria and the provision of a LLIN to every participant in the study. A recent study that examined the coverage of malaria control interventions in Burkina Faso reported that 59% of households in the study population owned an insecticide-treated bed net (ITN) and only 34% of children under 5 years of age with a reported malaria case were treated with artemisinin-based combination therapy (ACT) [17]. It is likely that receipt of a LLIN by every study participant increased their use in both study arms. Similarly the high level of general medical attention and easy availability of a high-quality ACT to treat confirmed malaria cases throughout the duration of the study could have reduced the parasite reservoir in both study populations. The potential beneficial impact of these factors on both arms of the study would make it more difficult to determine a benefit due to the treatment of asymptomatic carriers in the intervention arm.

Another possible explanation for the lack of effect on the incidence of symptomatic malaria with a parasite density of >5,000/μL in children could be that the clearance of asymptomatic infection increased their risk of contracting a symptomatic infection. It has been suggested that asymptomatic carriage might be a form of tolerance to *P*. *falciparum* infection, which protects against the development of clinical episodes [18]. However, it should be noted that this is only a hypothetical explanation; the heterogeneity of malaria transmission, specifically variations in local transmission intensity, may confound the data indicating that clearance of asymptomatic *P*. *falciparum* infection increases vulnerability to clinical episodes [19,20]. A number of studies have examined the impact of clearing asymptomatic infections on the risk of subsequent clinical episodes, but the data are so far inconclusive [21-24].

The second primary objective explored the impact of treating asymptomatic carriers on Hb levels. While the intervention resulted in a statistically significant greater increase in Hb in asymptomatic carriers < 5 years of age in the intervention arm than in the control arm, this improvement was not of a sufficient magnitude to be considered clinically meaningful. As the baseline Hb level in both populations was in the normal range, a large increase in Hb could not have been expected. In 2010, a study documented a mean Hb level of 8.7 g/dL in children (personal communication, Dr A Tiono). However, over the past 10 years Burkina Faso has adopted a number of WHO-recommended interventions including the distribution of free ITNs/LLINs to vulnerable populations, and mass administration of antihelminthic drugs [25]. The impact of these interventions is likely to have improved baseline Hb level in children in certain communities in Burkina Faso to the point at which it is now difficult to identify further improvement. A Cochrane Review of the impact of ITNs concluded that sleeping under an ITN improved Hb level in children by 1.7% packed cell volume [26].

## Conclusion

This study has shown that the systematic screening and treatment of asymptomatic carriers at the community level can reduce gametocyte carriage in a population. However, in this setting the impact of the intervention was not sustained. This indicates greater levels of parasite clearance are required to interrupt transmission in this study setting.

## Competing interests

AT, AO, and BO have received honoraria from Novartis Pharma AG, Basel, Switzerland to attend Advisory Board meetings to discuss this study and manuscript. GO’N is an employee of Novartis Pharma AG, AM is an employee of Novartis Healthcare Private Limited, and KH is an employee of Novartis Pharmaceuticals Corporation. AD, SC, AG, and SS declared no competing interests.

## Authors’ contributions

All authors were involved in the design of the study, data interpretation, and defining the content for and critically reviewing the manuscript. AT, AO, AD, SC, AG, and SS were involved in data collection, while AT, AO, BO, AM, GO’N, and KH conducted the data analysis. AT, AO, BO, AM, and KH were involved in writing the manuscript. All of the authors had full access to data in the study, discussed the results, reviewed the draft manuscript and agreed on the final version. AT, the corresponding author, had final responsibility for the decision to submit the manuscript for publication. Editorial assistance was provided by Louisa Reed from PreScript Communications, with funding from Novartis Pharma AG.
